# Ultrasound-Assisted Fermentation to Remove Cadmium from Rice and Its Application

**DOI:** 10.3390/molecules28104127

**Published:** 2023-05-16

**Authors:** Xiaotong Yang, Jie Yin, Yahui Guo, Hang Yu, Shaofeng Yuan, He Qian, Weirong Yao, Jiangfeng Song

**Affiliations:** 1 State Key Laboratory of Food Science and Technology, National Centre for Technology Innovation on Fast Biological Detection of Grain Quality and Safety, School of Food Science and Technology, Jiangnan University, Wuxi 214122, China; 2 Institute of Agro-Product Processing, Jiangsu Academy of Agricultural Sciences, Nanjing 210014, China

**Keywords:** rice, ultrasonic, *Lactiplantibacillus plantarum*, cadmium removal rate, rice noodles

## Abstract

Rice, which is a major part of the daily diet, is becoming more and more contaminated by cadmium (Cd). This study combined low-intensity ultrasonic waves with the *Lactobacillus plantarum* fermentation method and optimized this technique by a single-factor and response surface experiment, aiming to solve the practical problems that the current Cd removal methods for rice cannot address, due to the fact that they require a long time (nearly 24 h), which prevents meeting the rice production demands. The described technique required a short time (10 h), and the highest Cd removal reached 67.05 ± 1.38%. Further analysis revealed that the maximum adsorption capacity of *Lactobacillus plantarum* for Cd increased by nearly 75%, and the equilibrium adsorption capacity increased by almost 30% after the ultrasonic intervention. Additionally, a sensory evaluation and other experiments proved that the properties of the rice noodles prepared from Cd-reduced rice obtained by ultrasound-assisted fermentation were comparable to those of traditional rice noodles, indicating that this method can be used in actual rice production.

## 1. Introduction

The amount of Cadmium (Cd) pollution caused by human activities is on the rise, which means that crops grown in the soil are absorbing more Cd than before [1]. In China, this is a particularly big problem, with almost 2.0 × 10^7^ hm^2^ of cultivated land contaminated with heavy metals. This corresponds to around one-fifth of the total cultivated land area there. Cd pollution is the most dangerous metal pollutant, and the annual production of agricultural products with an excessive Cd content reaches 1.46 billion kg. Other countries such as Japan and Thailand are also facing serious Cd pollution issues, affecting rice crops in particular. As early as the 1950s, Japan had an incident of ‘pain disease’ caused by the consumption of rice with an excess Cd content [2]. Studies have shown that Cd can significantly harm the nervous system, kidneys, and liver in the human body [3,4,5].

Different countries and organizations have issued guidelines for limiting Cd in cereal products. The Codex Alimentarius Commission (CAC) stipulates that the Cd limit in brown rice, polished rice, and steamed rice (semi-cooked rice) is 0.4 mg/kg. This standard has been adopted by countries such as Japan and Thailand because of the high levels of Cd present in their soil and water. In contrast, countries that import rice or have lower Cd levels in their soil and water have stricter standards. For example, China and the European Union have set a limit of 0.2 mg/kg of Cd, while Russia, Australia, and New Zealand have set a limit of 0.1 mg/kg of Cd. It is not allowed to use rice with an excessive Cd content during food processing, which results in significant food waste and financial losses.

Rice needs to be undergo Cd removal before becoming a food raw material. However, in industrial production, soaking is not sufficient to eliminate Cd from rice that is severely polluted. Chemical methods can be more efficient, but they leave behind chemical residues, which is a problem when in food production [6]. One alternative is to use bacteria to absorb Cd. *Lactobacillus plantarum* (*L. plantarum*) and yeast are the most common Cd-removing fermentation strains [7]. A study used different microorganisms to ferment rice [8]. The results showed that *L. plantarum* exerted the best modification effect on rice noodles, mainly because of its high enzyme production and acid production capacity, and could significantly reduce protein and ash content during fermentation. Acid production by fermentation can promote the dissolution of proteins, especially lactic acid, with its α-hydroxyl structure, which can favor the continuous migration of organic and inorganic heavy metal ions into the fermentation broth. Diatomite co-immobilized *L. plantarum* pellets achieved a 90% Cd removal rate, but this method is complex and cannot be applied in traditional rice processing [9].

Traditionally, ultrasonication has been used in food processing for many years to inhibit microbial activity. Recent research has shown that when the intensity of ultrasonic waves is low (less than 1 W/cm^2^), ultrasonication can actually help microorganisms grow and reproduce, which enhances the efficiency of the fermentation process [2,10]. Studies even demonstrated that low-intensity ultrasonication can increase the biomass and metabolic level of *Saccharomyces cerevisiae* [11,12].

The goal of this study was to find a way to remove Cd from fermented rice faster and more effectively. The response surface optimization method was used to explore the best ultrasonic intervention conditions for Cd removal from fermented rice. At the same time, the effect of a low-intensity ultrasonic intervention on *L. plantarum* was studied to analyze the reasons for the increase in Cd removal rate that it produces. Finally, rice noodles were made from Cd-reduced rice obtained by ultrasound-assisted fermentation and compared with traditional rice noodles to explore the practical application value of this method.

## 2. Results

### 2.1. Growth Curve of L. plantarum and Results of Ultrasound Application on Its Growth

**Figure 1:**
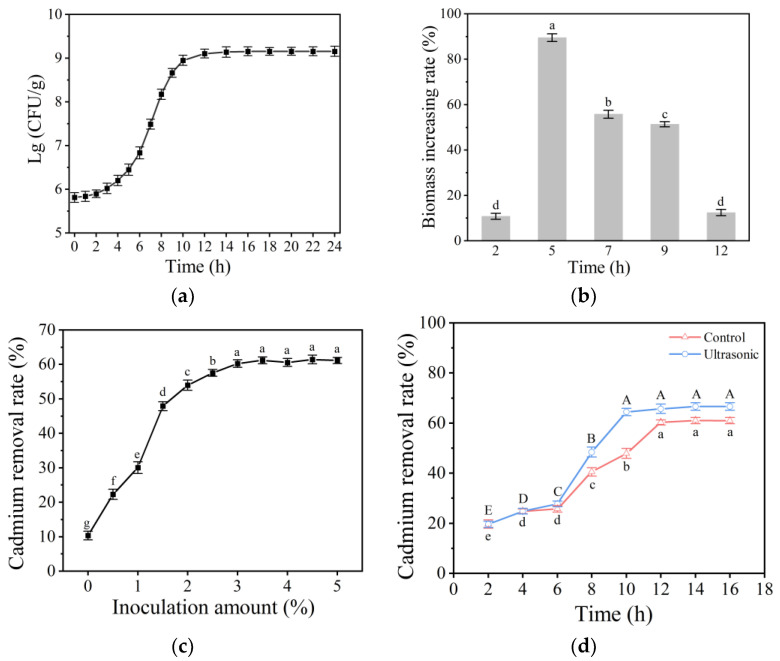
(**a**) Growth curve of *L. plantarum*; (**b**) Effect of ultrasonic treatment imposed at different growth phases on the biomass of *L. plantarum*; (**c**) Effect of inoculation amount and (**d**) fermentation time on Cd removal rate. (Different lowercase letters or uppercase letters in the figures represent significant differences, *p* < 0.05).

As shown in Figure 1a, the growth curve of *L. plantarum* is of S-type. In the beginning, from 0 to 3 h, growth was limited. Later, from 3 to 12 h, *L. plantarum* started growing and becoming more metabolically active. After 12 h, growth started to stabilize. A low-intensity ultrasonic treatment was performed on *L. plantarum* at different growth stages (Figure 1b). Compared with the control group, the intervention with low-intensity ultrasonication led to an increase in the biomass of *L. plantarum*. The growth-promoting effect was the best when ultrasonication was applied in the fifth hour, and the biomass of *L. plantarum* increased by 89.48 ± 1.37%. Therefore, the early exponential stage (5 h) was selected as the ultrasonic intervention stage.

### 2.2. Determination of Fermentation Conditions

The relationship between the inoculation amount of different *L. plantarum* and the Cd removal rate at the same fermentation time, is shown in Figure 1c. The Cd removal rate increased with the increase in inoculation amount until it reached the maximum at 3.0%. Therefore, 3.0% was chosen as the optimal inoculation amount for further research. Figure 1d displayed the impact of ultrasonication on Cd removal by *L. plantarum* during different fermentation times. The results indicated that it took 12 h to achieve the highest Cd removal rate without ultrasonic treatment, while the ultrasonic group achieved it in 10 h. Moreover, the Cd removal rate was approximately 7.6% higher than that of the control group, indicating that ultrasonic intervention had a significant effect on increasing the Cd removal rate (*p* < 0.05). As a result, 10 h was selected as the fermentation time.

### 2.3. Single Factor Experimental Results of Ultrasonic-Assisted Fermentation Method

**Figure 2:**
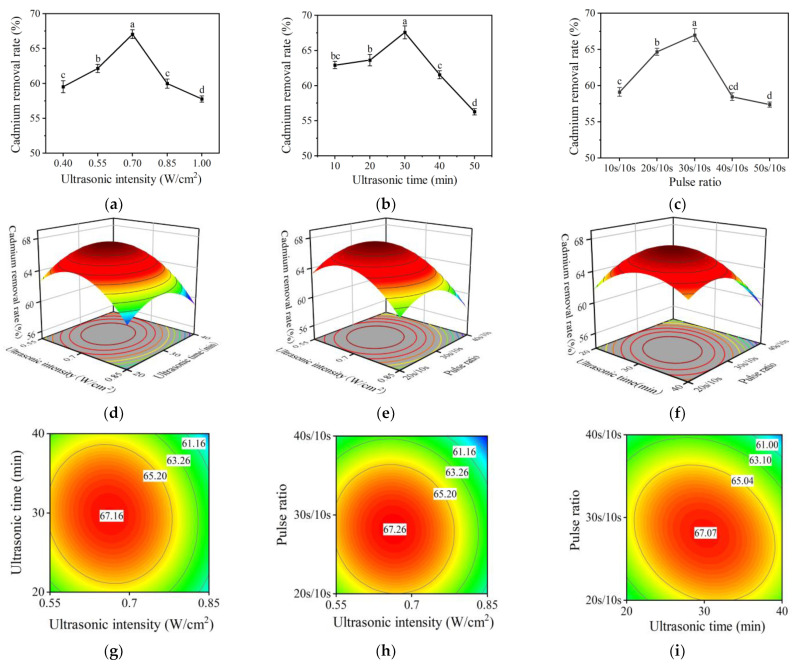
Effect of ultrasonic intensity (**a**), ultrasonic time (**b**), and pulse ratio (**c**) on the Cd removal rate; response surface diagram (**d**–**f**) and contour diagram (**g**–**i**) for the examined factors. (Different lowercase letters in the figures represent significant differences, *p* < 0.05).

According to the results shown in Figure 2a–c, the removal rate of Cd was affected by ultrasonic intensity, ultrasonic time, and pulse ratio. Figure 2a indicated that the Cd removal rate increased significantly within the range of 0.40–0.70 W/cm^2^ (*p* < 0.05), with the maximum rate (67.05 ± 0.62%) being achieved at 0.7 W/cm^2^. Figure 2b showed that the optimal ultrasonic time was 30 min, and the highest Cd removal rate was 67.57 ± 0.93%. Figure 2c showed that the optimal Cd removal rate of 66.96 ± 0.89% could be achieved at the pulse ratio of 30 s/10 s. Overall, the effects of three single factors on the Cd removal rate showed a trend of increasing first and then decreasing. This may be because within a specific range, with the increase of single-factor conditions, the growth and metabolism of *L. plantarum* were promoted.

### 2.4. Response Surface Test Results of the Ultrasoound-Assisted Fermentation Method

#### 2.4.1. Response Surface Design and Results

The response surface test was designed based on the results of single-factor experiments, and the scheme and results are shown in Table 1. Through multiple regression fitting, the regression equation was obtained:

Y = −61.258 + 201.957A + 2.037B + 2.183C − 0.296AB − 0.207AC − 0.0104BC − 141.080A^2^ − 0.0256B^2^ − 0.0307C^2^

The variance analysis of the regression equation is shown in Table 2. The *p*-value of the regression model was 0.0012 (<0.05), indicating that the regression was significant. The *p*-value of the lack of fit was 0.6608 (>0.05), i.e., not significant, indicating that the established quadratic regression model fitted well with the actual situation. This model could be used to analyze and predict the conditions for the ultrasound-assisted fermentation-mediated Cd removal process [13]. The correlation coefficient R^2^ was 0.9461, indicating that the regression equation could be used for the theoretical prediction of Cd removal from rice by ultrasound-assisted fermentation. The model adjustment coefficient R^2^_adj_ was 0.8767, meaning that the model could explain 87.67% of the change in the response value. The model fitted the test well, and the test error was small. C had a significant effect on the Cd removal rate for rice (*p* < 0.05), as well as A, A^2^, B^2^, and C^2^ (*p* < 0.01). According to the results, the primary and secondary factors affecting the Cd removal rate were A, C, and B, namely, ultrasonic intensity, pulse ratio, and ultrasonic time.

#### 2.4.2. Interaction Analysis

The response surface and contour results for various factors can be seen in Figure 2d–i. According to the obtained model, the optimal ultrasonic-assisted fermentation conditions that we predicted included ultrasonic intensity of 0.66 W/cm^2^, ultrasonic time of 30.22 min, and pulse ratio of 28.17 s/10 s, with the corresponding Cd removal rate of 67.26%. Considering the actual situation, the predicted optimal ultrasonic conditions were changed to ultrasonic intensity of 0.66 W/cm^2^, ultrasonic time of 30 min, and pulse ratio of 28 s/10 s. After the verification experiments, performed five times, it was found that the Cd removal rate under the optimal conditions was 67.05 ± 1.38%, which was close to the theoretical value. The model could predict the actual Cd removal bs ultrasound-assisted fermentation.

### 2.5. Effect of Low-Intensity Ultrasonication on L. plantarum

#### 2.5.1. Changes in Cell Membrane Permeability

**Figure 3:**
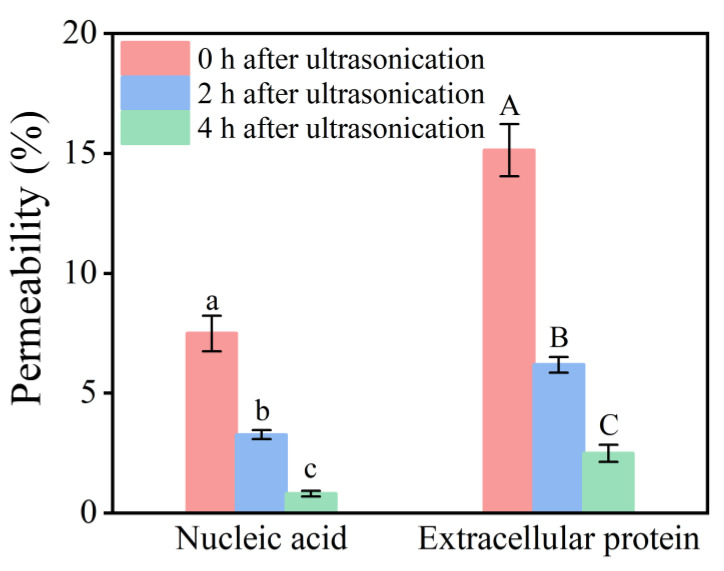
Effect of ultrasound on the cell membrane permeability of *Lactobacillus plantarum.* (Different lowercase letters or uppercase letters in the figures represent significant differences, *p* < 0.05).

Figure 3 shows that the ultrasonic intervention had an impact on the permeability of nucleic acids and extracellular proteins, which reflected changes in microbial cell membrane permeability. After the ultrasonic treatment, the nucleic acids and proteins in the cell membrane of *L. plantarum* had different degrees of exudation compared to their control levels. The nucleic acid permeability was 7.56 ± 0.73%, and the protein permeability was 15.16 ± 1.09%, meaning that low-intensity ultrasonication caused a local rupture of the cell membrane of *L. plantarum* and increased its permeability. With the prolongation of time, the permeability of nucleic acids and proteins gradually decreased, and the permeability of the cell membrane almost returned to the original state after 4 h, which proved that the rupture of the cell membrane was reversible. At the same time, our experiments [11] showed that this sublethal state could promote the exchange of substances between the inside and the outside the cell membrane, promote metabolical activity, and increase the total amount of bacteria. Therefore, it was speculated that this effect sustained the increase in *L. plantarum* biomass.

#### 2.5.2. Isothermal Adsorption Model of Cd

**Figure 4:**
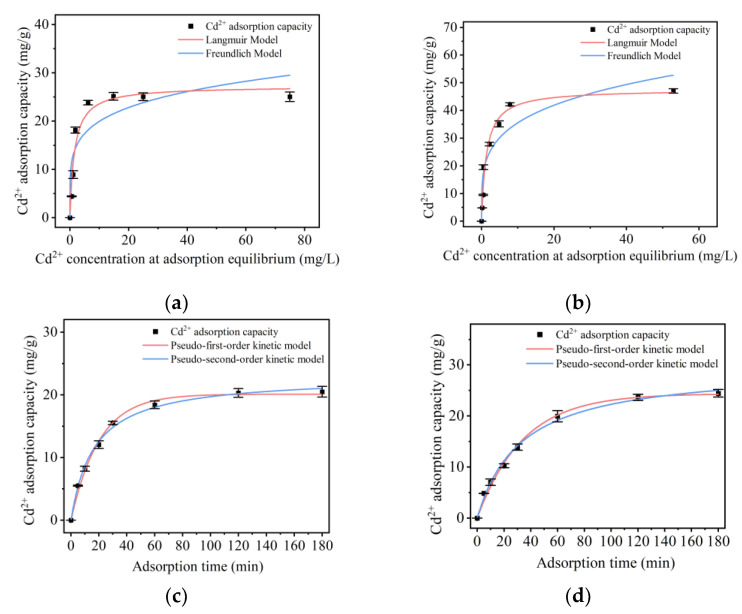
Isothermal adsorption model for control group (**a**) and ultrasound-subjected group (**b**); kinetic model for control group (**c**) and ultrasound-subjected group (**d**).

As shown in Figure 4a,b, the isothermal adsorption of Cd ions at different concentrations was studied in two groups of *L. plantarum* [14]. The results showed that with the initial Cd ion concentration increase, the adsorption efficiency increased first and then decreased. The Cd ion adsorption capacity of the control group (Figure 4a) was stable at about 30 mg/g, while that of the ultrasound-subjected group (Figure 4b) was stable at about 50 mg/g. To compare the results of the two groups more accurately, the Langmuir and the Freundlich models were used. The results are shown in Figure 4 and Table 3. The results showed that the adsorption of Cd by the two groups of *L. plantarum* was more consistent with the Langmuir model; that is, in both groups, monolayer adsorption occurred, and the distribution of the adsorption sites on the surface of the adsorbent was uniform. At the same time, compared with the control group, the maximum adsorption capacity (*Q_max_*) and adsorption coefficient (*b_L_*) of the ultrasound-subjected group increased by about 63% and 70%, respectively. This result indicated that the adsorption capacity of *L. plantarum* for Cd was improved after the ultrasonic treatment, which might be due to the significant increase in cell concentration and adsorption sites following the growth-promoting effect of ultrasonication.

#### 2.5.3. Adsorption Kinetics of Cd

Adsorption kinetics can be used to study the adsorption rate constant and adsorption control mechanism of Cd for *L. plantarum* [15]. Therefore, the adsorption kinetics of the two groups was studied. The fitting results of the quasi-first-order kinetic and quasi-second-order kinetic model are shown in Figure 4c,d and Table 4. The results showed that the adsorption of Cd by the two groups of *L. plantarum* was more in line with the pseudo-second-order kinetic model; that is, the rate-limiting step in the adsorption process could be chemical adsorption, complexation, or chelation involving valence forces through electron sharing or exchange between the bacterial adsorbent and the adsorbate. The quasi-second-order kinetic model fitting results showed that the control group’s equilibrium adsorption capacity (*Q_e_*) was 23.271 ± 0.558 mg/g, and the equilibrium adsorption capacity of the ultrasound-treated group was 26.666 ± 1.664 mg/g. The equilibrium adsorption capacity of *L. plantarum* for Cd increased by nearly 15% after ultrasonication, indicating that low-intensity ultrasonication promoted the growth and metabolism of *L. plantarum*, thereby promoting the adsorption of Cd ions. The pseudo-second-order adsorption rate constant (k_2_) for Cd adsorption by *L. plantarum* changed from 0.003 ± 0.0002 to 0.002 ± 0.0002 after ultrasonication. This may be because at the initial stage of the ultrasonic intervention, *L. plantarum* was in a sublethal state, and its adsorption capacity was limited. With time, its adsorption capacity gradually increased, and the cell concentration of Cd gradually rose. At 30–60 min, the equilibrium adsorption capacity exceeded that of the control group, verifying the conclusion of Section 2.4.1.

### 2.6. Quality Comparison of Rice Noodles

#### 2.6.1. Cooking Quality and Texture Quality

**Figure 5:**
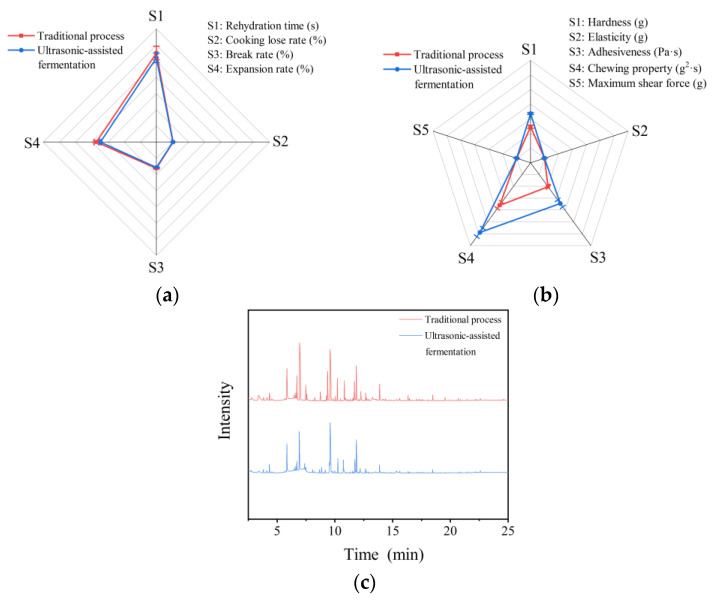
Cooking quality (**a**) and texture quality (**b**) of two kinds of rice noodles; (**c**) gas chromatogram of the two kinds of rice noodles.

As shown in Figure 5a, the cooking quality of the two kinds of rice noodles had little differences. There was no significant difference in the rehydration time, cooking loss rate, break rate, and expansion rate of the rice noodles (*p* > 0.05). Figure 5b showed the texture quality results of the two types of rice noodles. There was no significant difference in the elasticity of the two types of rice noodles (*p* > 0.05), indicating that the ultrasound-assisted fermentation method did not affect their elasticity. After the Cd removal treatment through ultrasound-assisted fermentation, the rice noodles’ adhesiveness and chewing properties increased, probably because holes were formed on the surface and inside the grains when Cd was removed from the rice. In processing the rice noodles, starch molecules were more likely to develop a dense gel network [16]. Therefore, the ultrasound-assisted fermentation method allowed retaining the original quality of the rice noodles and gave them a new taste, while achieving a high Cd removal.

#### 2.6.2. Volatile Components

Figure 5c and Table A1 illustrate the volatile components analysis results of the two types of rice noodles. The volatile components discovered in both types of noodles were alcohols, esters, aldehydes, ketones, acids, and aromatic compounds. The main odor of traditional rice noodles is the almond aroma, followed by a fruit aroma, accompanied by a certain wine aroma and vinegar aroma. Compared with the traditional rice noodles, the almond flavor was less intense after the rice noodles were treated with ultrasound-assisted fermentation, the wine flavor disappeared, and the fruit flavor became predominant. This means that ultrasound-assisted fermentation may improve the flavor of rice noodles and has application potential.

#### 2.6.3. Sensory Evaluation Analysis

Table 5 is a summary of the sensory evaluation data of the two types of rice noodles. The fuzzy matrices R_1_ and R_2_ of two rice noodles were obtained by using the fuzzy mathematics method.

$$R_{1}=\left[\begin{array}{ccccc} 0.55 & 0.35 & 0.10 & 0 & 0\\ 0.55 & 0.25 & 0.20 & 0 & 0\\ 0.60 & 0.35 & 0.05 & 0 & 0\\ 0.60 & 0.25 & 0.15 & 0 & 0\\ 0.55 & 0.30 & 0.15 & 0 & 0 \end{array}\right],\;R_{2}=\left[\begin{array}{ccccc} 0.55 & 0.30 & 0.15 & 0 & 0\\ 0.50 & 0.30 & 0.20 & 0 & 0\\ 0.45 & 0.30 & 0.25 & 0 & 0\\ 0.50 & 0.25 & 0.25 & 0 & 0\\ 0.60 & 0.35 & 0.05 & 0 & 0 \end{array}\right]$$

From the comprehensive evaluation set Y = XR and weight set $X=\left(\begin{array}{ccccc} 0.23 & 0.20 & 0.20 & 0.17 & 0.20 \end{array}\right)$, the complete evaluation set of the two kinds of rice noodles was obtained: $Y_{1}=\left(\begin{array}{ccccc} 0.6555 & 0.30 & 0.13 & 0 & 0 \end{array}\right)$, $Y_{2\;}=\left(\begin{array}{ccccc} 0.598 & 0.30 & 0.18 & 0 & 0 \end{array}\right)$. According to the five grades of each evaluation index, A, B, C, D and E, which correspond to 100, 80, 60, 40 and 0 points, the total scores for the two samples were 97.35 and 94.60. The total score of the ultrasound-assisted fermentation-treated rice noodles was close to that of the traditionally processed rice noodles. This indicated that the ultrasound-assisted fermentation did not destroy the texture of the rice noodles and affected their smoothness, gumminess, and color less than the traditional process. In terms of flavor, the rice noodles after ultrasound-assisted fermentation had a better flavor than the traditional rice noodles, which may be because of the effect of *L. plantarum*. This also directly proved that ultrasound-assisted fermentation allowed retaining the original quality of the rice noodles, while improving the efficiency of Cd removal and may thus have application value.

## 3. Discussion

Previous works used different methods to efficiently remove Cd from rice flour, without focusing on reducing Cd from whole rice. Several techniques exist for removing cadmium from rice [17]. The cleaning method is the most convenient for Cd removal, but its Cd removal rate is only 1–8% [18]. The Cd removal rate of the soaking method can reach about 60%, but this methos needs a 47 h soaking time, resulting in the loss of rice quality [19]. At the same time, a study showed that the cadmium removal rate was only about 4% when using short-time soaking (1–8 h) [20]. Starch extraction from Cd-contaminated rice was carried out using the alkali method. The finished starch had a 60% decrease in Cd content compared to the raw material [21]. The highest Cd removal rate, up to 92%, was achieved using hydrochloric acid or an EDTA disodium solution to soak the rice proteins [22]. However, chemical reagents cannot be used in the actual production of food due to their residues remaining in the treated material. Biological methods are safe, with a high Cd removal rate. In this case, Cd reduction involves mainly microbial fermentation, adsorption of cadmium and its metabolites, separation of cadmium from proteins, or promotion of the dissolution of cadmium-binding proteins to reduce cadmium in the system [23,24]. Studies have shown that *L. plantarum* fermentation achieved more than 80% Cd removal [7,9,25]. However, in the production process, it is generally necessary to control the pretreatment time, maintaining it within 12 h. The fermentation method based on *L. plantarum* is limited by the growth of *L. plantarum* in the early stage of fermentation; therefore, the Cd removal rate can only reach about 35% in the early stage of fermentation (0–10 h). This method needs approximately 24 h to achieve the maximum Cd removal rate; thus, it is still difficult to apply it in food processing. Only a few authors examined the combination of several methods of Cd removal [26].

Our study presents a highly effective approach for the removal of Cd by combining physical and biological methods. In this study, a prediction model of the Cd removal rate of ultrasound-assisted fermentation was stablished to obtain the optimal conditions. The attained results suggested that low-intensity ultrasonication had a growth-promoting effect on *L. plantarum*, especially in its early stage of growth. Additionally, the isothermal adsorption model and kinetic model showed that the adsorption capacity of *L. plantarum* for Cd was significantly improved after the ultrasonic intervention. These phenomena might contribute to the efficient Cd removal by the ultrasonic-assisted fermentation method.

The Cd-reduced rice noodles obtained by ultrasonic-assisted fermentation had similar cooking quality and elasticity as the traditional rice noodles. The sensory evaluation experiment showed that the ultrasound-assisted fermentation-treated rice noodles would lose some smoothness and color and become sticky. Still, the method improved their smell while retaining their texture, which indicates that the ultrasound-assisted fermentation method has interesting application potential, while achieving efficient Cd removal. In this study, the method was used especially for Cd-contaminated rice, allowing a much more meaningful application in comparison to traditional procedures. Therefore, it could represent an innovation allowing the safe use of Cd-contaminated rice resources.

Nevertheless, further studies are necessary to develop a new optimized method, which will consider the promoting effect of low-intensity ultrasonication on various microorganisms and the synergistic effect between different microorganisms. Among our achievements, we were able to shorten the time of the Cd removal process.

## 4. Materials and Methods

### 4.1. Experimental Strain and Raw Materials

*Lactiplantibacillus plantarum* CICC20261 was purchased from the China Center of Industrial Culture Collection (Beijing, China). The MRS broth (de Man, Rogosa and Sharpe Broth) was purchased from Beijing Land Bridge Technology Co., Ltd. (Beijing, China). Nitric acid for pure analysis (≥60–65%) was purchased from Sinopharm Chemical Reagent Co., Ltd. (Shanghai, China). The Cd standard stock solution and internal standard indium stock solution (the concentration of the elements was 100 μg/mL, and that of the substrate was 0.1% HNO_3_) were purchased from China Grinm Group Co., Ltd. (Beijing, China). Rice (the content of Cd was 0.239 ± 0.040 mg/kg) was produced in Hunan Province (China). Ultrapure water was processed through a milli-Q ultrapure water preparation instrument Merck Co., Ltd. (Darmstadt, Germany).

### 4.2. Method for the Determination of the Cd Content

**Figure A1:**
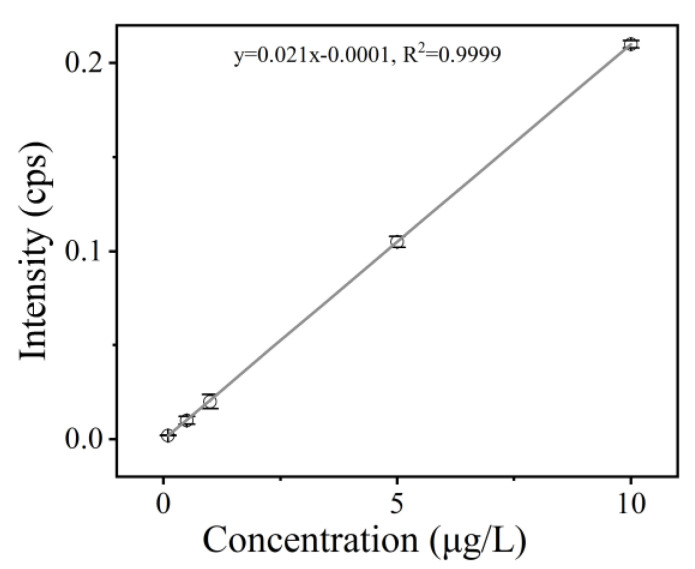
Calibration curve constructed by plotting Cd concentration versus ICP-MS intensity.

An Inductively Coupled Plasma Mass Spectrometer (NEXION350D, Perkin Elmer Ltd., Waltham, MA, USA) and a Microwave Digestion Instrument (ETHOSUP, Milstone Co., Ltd., Shelton, CT, USA) were used to measure the Cd content based on a previous study [27]. The standard curve is shown in Figure A1, R^2^ = 0.9999, and the limit of quantification was 0.1 μg/L Cd. The samples were spiked with 0.2, 1, and 2 mg/kg of Cd, and the recoveries were 98.90%, 101.35%, and 102.77%, respectively. The uncertainty was 0.0101 [28]. This result indicated that the standard curve could determine the concentration of samples in this range. Briefly, a dried sample of 0.200 g (accurate to 0.001 g) was weighed in the microwave digestion inner tank, and 5.0 mL of HNO_3_ was added. After overnight incubation, the digestion was carried out according to the microwave digestion instrument program. The tank was removed, and the lid was opened after the tank was cooled. The digestion tank was placed on a temperature-controlled electric heating plate at 100 °C for 30 min, then water was added up to 50.0 mL to dilute the sample, and a control group was set. The Cd content was calculated according to Equation (1):

(1) $$X=\frac{\left(\rho -\rho_{0}\right)\times V\times f}{m\times 1000}$$

where *X* is the content of Cd in the sample (mg/kg); *ρ* is the mass concentration of Cd in the sample solution (μg/L); *ρ*_0_ is the mass concentration of Cd in the blank solution (μg/L); *V* is the constant volume (mL) of the sample digestive fluid; *f* is the dilution multiple of the sample; *m* is the sample weighing quality (g); 1000 is the conversion coefficient.

### 4.3. Activation of L. plantarum and Growth Curve Determination

For the activation and growth curve determination methods for *L. plantarum*, we referred to previous research methods [29,30]. Under sterile conditions, the freeze-dried strain powder was activated in MRS broth and inoculated obliquely. The activated bacterial solution was packaged with 50% glycerol and stored in a freezer at −80 °C. A ring of colonies was taken from the surface of the inclined medium in an ultra-clean bench and inoculated into the liquid medium. The seed liquid was prepared statically at 37 °C for 12 h. The freeze-dried powder of *L. plantarum* was converted into seed liquid after activation and culture. Then, 3.0% (*v*/*v*) of the seed liquid, after dilution to 10^8^ CFU/mL (OD_600_ = 0.3) by the MRS broth, was inoculated in MRS broth and incubated at 37 °C to determine the growth curve. Samples were taken at one-hour intervals between 0 and 10 h and every two hours between 10 and 30 h of incubation. The absorbance at 600 nm (A_600_) of samples at each time point was measured by a UV–visible spectrophotometer (UV-1800, Shimadzu Co., Ltd., Kyoto, Japan). MRS medium was used as the control group, and each sample was diluted with MRS medium to control the determination results of the diluted samples between 0.2 and 0.8 absorbance. The sample volume was 3 mL, the optical path was 10 mm, and the detection wavelength was 600 nm.

### 4.4. Determination of the Ultrasonic Intervention Stage

According to its growth curve, *L. plantarum* was incubated at 10^8^ CFU/mL, 3.0% (*v*/*v*), and 37 °C. Ultrasonication was applied at different stages of its growth by an industrial ultrasonic processor (UIP500hdT, Hielscher GmbH, Teltow, Germany) [31]. The ultrasonic intensity of 0.7 W/cm^2^, the ultrasonic time of 30 min, and the pulse ratio of 30 s/10 s (ultrasonic opening 30 s, closing 10 s) were selected as the ultrasonic conditions, and a group without ultrasonic treatment was set as the control group.

### 4.5. Determination of the Fermentation Conditions

Referring to the methods of a previous study [32], Cd-contaminated rice was mixed with ultrapure water at a ratio of 1: 4 (*w*/*v*), then inoculated with 0, 0.5, 1.0, 1.5, 2.0, 2.5, 3.0, 3.5, 4.0, 4.5, and 5.0% (*v*/*v*) of the *L. plantarum* suspension and cultured at 37 °C for 12 h, in the dark in a constant-temperature foster box, without stirring. The Cd removal rate of each sample was determined to determine the appropriate inoculation amount of *L. plantarum*. It can be seen in Section 2.3 that 3.0% of bacterial suspension was selected for the subsequent experiments. Then, the ultrasonic treatment was performed with the appropriate inoculation amount, and the incubation was continued. Samples were taken every 2 h to measure the Cd removal rate at each time point. The non-ultrasonic treatment group in the same conditions was set as the control group.

### 4.6. Single-Factor Experiment of Ultrasonic Assisted Fermentation

The diluted seed liquid of *L. plantarum* was inoculated into a rice soaking solution (*w*/*v* = 1:4) at 10^8^ CFU/mL and 3.0% (*v*/*v*). During the fermentation process at 37 °C, different ultrasonic conditions were applied. The ultrasonic intensity was 0.40, 0.55, 0.70, 0.85, and 1.00 W/cm^2^, the ultrasonic time was 10, 20, 30, 40, and 50 min, and the pulse ratio was 10 s/10 s, 20 s/10 s, 30 s/10 s, 40 s/10 s, and 50 s/10 s. A thermostatic circulator was used to keep the temperature constant during the ultrasonic process. The fermentation time was 10 h. After this time, the rice was separated and stored in a refrigerator at 4 °C for the subsequent determination of its Cd content.

### 4.7. Response Surface Experiment of Ultrasonic Assisted Fermentation

A three-factor and three-level response surface test was designed by Design-expert software, with Cd removal rate as the dependent variable and ultrasonic intensity, ultrasonic time, and pulse ratio (shown in Table 6) as independent variables, based on the single-factor experiment results. Seventeen groups were analyzed to detect the Cd removal rate under different conditions.

The results of the response surface test were subjected to variance analysis. Then, a reliable prediction model was established, and the experiments were repeated five times to predict and verify the ultrasonic-assisted fermentation conditions with the best Cd removal rate.

### 4.8. Research Methods to Determine the Cell Membrane Permeability of L. plantarum

The changes in the cell membrane permeability of *L. plantarum* within 0, 2, and 4 h after the intervention with the best ultrasonic conditions (0.66 W/cm^2^, ultrasonic time 30 min, pulse ratio 28 s/10 s) were studied, using a method described in a previous study [33]. The samples without ultrasonic treatment were used as a control.

### 4.9. Study of the Adsorption of Cd by L. plantarum

Rice noodles were produced by a stainless-steel rice noodles machine (SZ-150, Xuzhong Food Machinery Co., Ltd., Guangzhou, China). The study was based on an isothermal adsorption model and adsorption kinetics [34]. The adsorption of Cd by *L. plantarum* after ultrasonic treatment was fitted, and the adsorption parameters were calculated. Samples without ultrasonic treatment under the same conditions were used as a control.

### 4.10. Determination Method for the Cooking Quality of the Rice Noodles

For the determination of the cooking quality, we referred to a previous study [35]. The rehydration time, cooking loss rate, break rate, and expansion rate of traditional rice noodles and ultrasound-assisted fermentation-treated rice noodles were compared. We put 5.0 g of rice noodles into 50 mL (*m*/*v* = 1:10) of boiling water for 10 min, then took a rice noodle every 30 s and placed it between two glass plates for extrusion; we observed whether there was a white hardcore, and recorded the soaking time in hot water when no hardcore was present, that is, the rehydration time. We selected 150.0 g of rice noodles that were more than 20 cm in length. The cooking time was 1 min longer than the rehydration time. Supercooled water was dried after 1 min. Rice noodles less than 10 cm long were separated from those that were more than 10 cm long. The rice noodles were weighed, and their weights were recorded as *m*_1_ and *m*_2_; the broken rate was calculated according to Equation (2). We took then 5 g of rice noodles; after a cooking time 1 min longer than the rehydration time, the drain water was removed. The quality of the rice noodles was recorded as *m*_3_. After drying, the quality of the rice noodles was recorded as *m*_4_, and the expansion rate was calculated according to Equation (3). After the rice noodle soup was stirred evenly, a tenth of the volume of the soup was taken and put into a beaker (dried) to dry until a constant weight was recorded as *m*_5_; the mass of the beaker was *m*_6_, and the cooking loss was calculated according to Equation (4).

(2) $$\mathrm{Cooking}\;\mathrm{lose}\;\mathrm{rate}\;(\%)=\;\frac{m_{1}}{m_{1}+m_{2}}\;\times \;100\%$$

(3) $$\mathrm{Expansion}\;\mathrm{rate}\;(\%)\;=\;\frac{m_{3}-m_{4}}{m_{4}}\;\times \;100\%$$

(4) $$\mathrm{Break}\;\mathrm{rate}\;(\%)\;=\;\frac{m_{5}-m_{6}}{m_{4}}\;\times \;10\;\times \;100\%$$

### 4.11. Determination Method of the Texture Quality of the Rice Noodles

The texture quality of the two rice noodle samples after cooking was determined by a physical property analyzer (TA-XT plus, Stable Micro System, Godalming, UK) based on a previous study [36]. To conduct the test, the noodles were cooked and cut into 5 cm pieces. A single noodle strand was then securely fastened with adhesive tape to the smooth platform (Base TA-90) of the analyzer. The noodle was subjected to a deformation test in a TPA cycle using a cylindrical probe (38 mm) at a speed of 1.0 mm/s.

The settings used for the test were as follows: Mode: Texture profile analysis; Option: Return to start; Pre-Test Speed: 2.0 mm/s; Test Speed: 1.0 mm/s; Post-Test Speed: 2.0 mm/s; Strain: 75% deformation in compression mode; Stationary Time: 1 s; Trigger Force: 0.1 N; Load Cell: 25 kg; Data Acquisition Rate: 20 pps.

### 4.12. Determination Method of the Flavor of the Rice Noodles

According to the methods reported in a previous study [37], the flavor of the rice noodles was determined by a Benchtop Gas Chromatography–Time-of-Flight Mass Spectrometer (Pegasus BT, LECO, Saint Joseph, MO, USA). The mass of the rice noodles in a sample bottle was 5.00 g. A DB-5 MS column (30 m × 0.25 mm × 0.25 μm) was used. Temperature programming: the initial temperature was 40 °C, maintained for 3 min, then increased to 250 °C at 10 °C/min and maintained for 6 min. The transmission line temperature was 250 °C. The electron impact source was selected. The ion source temperature was 210 °C. The mass scanning range was 35–400 amu. Helium gas was the carrier gas, while the constant current mode was used. The flow rate was 1.0 mL/min, without split injection.

### 4.13. Sensory Evaluation Method of the Rice Noodles

The cooked rice noodles were evenly divided and comprehensively evaluated for five aspects: texture, smoothness, adhesion, color, and flavor [38]. Each evaluation index assigned grades, i.e., A, B, C, D, and E, corresponding to 100, 80, 60, 40, and 0 points, respectively. The sensory evaluation criteria for the rice noodles are shown in Table 7. The fuzzy mathematics comprehensive evaluation method was used to analyze the evaluation table.

### 4.14. Data Analysis

All experiments were repeated at least three times. SPSS 26 software was used for data analysis, and the results are expressed as mean ± standard deviation. One-way analysis of variance (ANOVA) was used to analyze the correlation between the data, and *p* < 0.05 was considered statistically significant. The response surface test and response surface analysis were designed by Design-expert 13 software. Drawing was conducted with Origin 2022.

## Figures and Tables

**Table 1:** Response surface test design.

	A Ultrasonic Intensity (W/cm^2^)	B Ultrasonic Time (min)	C Pulse Ratio	Y Cd Removal Rate (%)
1	−1 (0.55)	−1 (20)	0	62.004
2	−1	0 (30)	−1 (20 s/10 s)	63.022
3	−1	1 (40)	0 (30 s/10 s)	63.656
4	−1	0	1 (40 s/10 s)	61.580
5	1 (0.85)	−1	0	59.679
6	1	0	−1	60.471
7	1	1	0	59.558
8	1	0	1	57.786
9	0 (0.70)	0	0	65.600
10	0	0	0	67.816
11	0	0	0	68.046
12	0	0	0	65.568
13	0	0	0	67.774
14	0	−1	1	61.806
15	0	1	−1	62.924
16	0	−1	−1	62.356
17	0	1	1	58.220

**Table 2:** Analysis of variance.

Source	Sum of Squares	df	Mean Square	F-Value	*p*-Value
Model	159.71	9	17.75	13.64	0.0012
A-Ultrasonic intensity	20.37	1	20.37	15.66	0.0055
B-Ultrasonic time	0.28	1	0.28	0.21	0.6590
C-Pulse ratio	11.00	1	11.00	8.46	0.0227
AB	0.79	1	0.79	0.60	0.4623
AC	0.39	1	0.39	0.30	0.6027
BC	4.31	1	4.31	3.31	0.1115
A^2^	42.43	1	42.43	32.61	0.0007
B^2^	27.64	1	27.64	21.25	0.0025
C^2^	39.74	1	39.74	30.54	0.0009
Residual	9.11	7	1.30		
Lack of Fit	2.75	3	0.92	0.58	0.6608
Pure Error	6.36	4	1.59		
Cor Total	168.82	16			
		R^2^ = 0.9461	R^2^_adj_ = 0.8767		

**Table 3:** Fitting parameters of different isothermal adsorption models.

Isothermal Adsorption Model	Blank		Ultrasonication	
	Adsorption Coefficients	Fitting Degree (R^2^)	Adsorption Coefficients	Fitting Degree (R^2^)
Langmuir ($q_{e}=\frac{Q_{max}b_{L}C_{e}}{1+b_{L}C_{e}}$)	*Q_max_* = 30.840 ± 3.718	0.943	*Q_max_* = 49.130 ± 2.917	0.986
	*b_L_* = 0.321 ± 0.068		*b_L_* = 0.630 ± 0.055	
Freundlich ($q_{e}=K_{F}C_{e}^{1/n_{F}}$)	*K_F_* = 6.473 ± 1.106	0.889	*K_F_* = 11.022 ± 1.197	0.933
	*n_F_* = −0.409 ± 0.072		*n_F_* = −0.427 ± 0.045	

**Table 4:** Fitting parameters of different adsorption kinetic models.

Adsorption Kinetics Model	Blank		Ultrasonication	
	Adsorption Coefficients	Fitting Degree (R^2^)	Adsorption Coefficients	Fitting Degree (R^2^)
Pseudo-first-order kinetic model [ln(*q*_e_ − *q*_t_) = ln*q*_e_ − *k_1_t*]	*Q_e_* = 18.605 ± 0.774	0.997	*Q_e_* = 21.995 ± 2.012	0.997
	*k_1_* = 0.068 ± 0.005		*k_1_* = 0.049 ± 0.005	
Pseudo-second-order kinetic model [*t*/*q*_t_ = 1/*k_2_*^2^*q*_e_^2^ + *t*/*q*_e_]	*Q_e_* = 23.271 ± 0.558	0.999	*Q_e_* = 26.666 ± 1.664	0.999
	*k_2_* = 0.003 ± 0.0002		*k_2_* = 0.002 ± 0.0002	

**Table 5:** Sensory Index of the two kinds of rice noodles.

Rice Noodle	Sensory Index	A	B	C	D	E
Traditionally processed rice noodle	Chewiness	11	7	2	0	0
	Smoothness	11	5	4	0	0
	Gumminess	12	7	1	0	0
	Color	12	5	3	0	0
	Flavor	11	6	3	0	0
Ultrasound-assisted fermentation-treated rice noodle	Chewiness	11	6	3	0	0
	Smoothness	10	6	4	0	0
	Gumminess	9	6	5	0	0
	Color	10	5	5	0	0
	Flavor	12	7	1	0	0

**Table 6:** Levels of the three factors examined.

Factor	Level		
	−1	0	1
A-Ultrasonic intensity (W/cm^2^)	0.55	0.70	0.85
B-Ultrasonic time (min)	20	30	40
C-Pulse ratio	20 s/10 s	30 s/10 s	40 s/10 s

**Table 7:** Sensory evaluation of the two kinds of rice noodles.

Sensory Index (Weight)	Standard	Evaluation
Chewiness (0.23)	Chewy and moderate hardness	A, B, C, D, E
Smoothness (0.20)	Taste smooth	A, B, C, D, E
Gumminess (0.20)	Not sticky or half-cooked	A, B, C, D, E
Color (0.17)	White, uniform color, not variegated	A, B, C, D, E
Flavor (0.20)	Rice aroma and no peculiar smell	A, B, C, D, E

## Data Availability

The authors can provide the data if needed.

## Sample Availability

Samples of the compounds are available from the authors.

## Appendix A

**Table A1:** Analysis of volatile components.

CAS	Name	Formula	Odor Characteristic	Relative Content (%)	
				Traditional	Ultrasonic-Assisted Fermentation
106-42-3	p−Xylene	C_8_H_10_	Almond	4.052	5.911
95-47-6	o−Xylene	C_8_H_10_		1.778	−
540-18-1	Butanoic acid, pentyl ester	C_9_H_18_O_2_		13.459	−
98-01-1	Furfural	C_5_H_4_O_2_		5.412	−
108-05-4	Acetic acid ethenyl ester	C_4_H_6_O_2_	Fruit	2.090	3.110
66-25-1	Hexanal	C_6_H_12_O		5.432	9.615
71-41-0	1−Pentanol	C_5_H_12_O		0.900	1.427
124-13-0	Octanal	C_8_H_16_O		0.488	1.066
513-86-0	Acetoin	C_4_H_8_O_2_		4.462	3.346
111-27-3	1−Hexanol	C_6_H_14_O		2.231	3.508
64-19-7	Acetic acid	C_2_H_4_O_2_		2.620	4.647
124-19-6	Nonanal	C_9_H_18_O	Citrus	2.156	3.733
112-31-2	Decanal	C_10_H_20_O		1.244	1.272
100-52-7	Benzaldehyde	C_7_H_6_O	Cherry	0.791	1.096
64-17-5	Ethanol	C_2_H_6_O	Wine	3.361	−
100-41-4	Ethylbenzene	C_8_H_10_	Spicy	1.300	−
111-13-7	2−Octanone	C_8_H_16_O	Milk	0.048	1.054
107-92-6	Butanoic acid	C_4_H_8_O_2_	Cheese	2.519	0.519
108-24-7	Acetic anhydride	C_4_H_6_O_3_	Vinegar	6.837	3.284
3913-02-8	1−Octanol, 2−butyl−	C_12_H_26_O	Herb	1.613	−
